# News and Notes

**Published:** 1904-01

**Authors:** 


### *NEWS AND NOTES.*

It will be of interest to all physicians in the State to learn that a professional nurses' register has been established at Palmer's Viaduct Pharmacy, 11 Peachtree street, Atlanta. Only graduates and competent names are on this register, and one can be obtained by either writing, telegraphing or telephoning them. Phone 64.

---

Plague at Rio de Janeiro is reported to the extent of 29 deaths and 52 new cases, and 109 cases under treatment.

---

The State Board of Health of Indiana has issued orders forbidding the reemployment of 250 teachers in the public schools who are afflicted with tuberculosis.

---

It is reported that cholera has broken out at Bethlehem, and the town has been surrounded by a cordon of troops in the hope of preventing the invasion of Jerusalem by the disease.

---

The cornerstone of King Edward the Seventh Consumption Sanatorium was laid November 3 at Midhurst, Sussex, Eng., by the king. One million dollars was given for this by Sir Ernest Cassel.

---

Dr. M. V. Robinson, of Atlanta, has been appointed a member of the board of eclectic examiners by Governor Terrell for a term of three years. The board will meet at an early date to conduct the examination.

---

Dr. Nicholas Senn, who was recently taken seriously ill with septicemia in Kansas City, from an infected operation wound of the right hand, has returned to Chicago and is reported much improved and progressing rapidly toward recovery.

*Archives Latines de Medicine et de Biologie* is the title of a new trilingual monthly medical review, the first number of which was announced for publication on October 15. It will be published in French, Spanish, and Italian.

---

DR. E. R. ANTHONY, of Spalding county, has been named as a member of the alopathy board of medical examiners by Governor Terrell. Dr. Anthony is one of the leading physicians of Spalding county. His term is for three years from January 1.

---

DR. W. ROYAL STOKES, city bacteriologist, in a recent address said that since the introduction of the public distribution of diphtheria antitoxin the rate of mortality from this disease in Baltimore has fallen from 50 per cent. in 1894 to 13 per cent. at the present time.

---

CHAPTER 135 of the laws of Utah of 1903 provides that any person under the age of eighteen years who shall buy, accept or have in his possession any cigar, cigarette or tobacco in any form, or any opium or any other narcotic in any form, shall be guilty of a misdemeanor.

---

IN Italy as elsewhere the medical profession is overstocked, and the number of practitioners is steadily increasing. The number at the present time is given as 22,139, as against 18,984 in 1882. Of medical women there are now twenty, while in 1882 there were only two.

---

FRANCIS M. FRENCH, aged sixty-four, has taken the State medical examinations which are being held at Syracuse. He is thought to be the oldest candidate who has ever appeared in the city. Mr. French was sixty years old when he was graduated from the University of Denver.

THE French Academy of Medicine has awarded the prize of 100,000 francs ($20,000) for the most remarkable scientific work to Dr. Roux, of the Pasteur Institute. Dr. Roux accepted the prize, but requested that he be allowed to devote the amount to investigations at the Pasteur Institute.

---

PLAGUE is still causing death in Manila, notwithstanding the board of health is exercising every precaution to check its spread. The cargoes of all vessels entering and leaving this port are fumigated for the purpose of killing rats and vermin, which are thought to be the cause of the infection. A bonus of five cents is paid for every rat delivered to the health department.

---

AN unusual syphilitic lesion is recorded in the case of a pedestrian who was accidentally hit on the nose by the tip of a whip of a passing teamster. A typical chancre developed at the point of the slight excoriation, and investigation revealed that the teamster had syphilitic lesions in his mouth and was in the habit of nibbling his whiplash. This occurrence was reported by Dr. Melot in a French exchange.

---

PROF. JOHAN VON MIKULICZ-RADECKI, lecturing at Breslau recently on his recent trip to the United States, said that he found more fruitful ideas among the American surgeons than among the French and English. He added: "The time is past when we were the givers and Americans the receivers. The American character has as a fundamental feature unlimited self-confidence, and the American believes he can do anything he wishes."

---

IT will be remembered that Arthur C. Probert, vice-president of the Christian Hospital, was arrested on the complaint of Dr. J. B. Murphy and others on the ground that their names were being used without authority, on certificates of the hospital to induce other physicians to become members of the staff on payment of fees of varying amount. Probert was fined late in November $500 and costs for the fraudulent use of the mails.

---

AN opinion in a case was rendered recently which established the legal right of Christian Scientists to treat diseases of all kinds without danger of civil or criminal liability so long as they can confine themselves to Christian Science practice. In the same opinion, however, the court held that there was evidence upon which a jury might properly find that a defendant had deceived the plaintiff as to his injury.

---

THE German charlatan Runge has been condemned to three months' imprisonment at Lauenburg on account of the death of a young woman whom he treated by extensive venesection and application of twenty-two leeches. She had a gastric ulcer, and succumbed within a few days to debility from the loss of so much blood. Another charlatan, Scheibler, has been fined $75 at Berlin for fraudulent practices and the use of the title professor.

---

THE American Public Health Association, at its recent meeting in Washington, D. C., elected the following officers: President, Dr. Carlos J. Findlay, of Havana, Cuba; First Vice-President, Dr. J. R. Monjaras, of Mexico City; Second Vice-President, Dr. William C. Woodward, of the District of Columbia; Secretary, Dr. Charles O. Prabst, of Columbus, O.; Treasurer, Dr. Frank W. Wright, formerly city health officer of New Haven, Conn.

---

SEVERAL physicians of St. Louis, Mo., are considering the expediency of establishing a dairy in that city for the sale of goat's milk, which is said to be immune from tuberculous qualities and to be easily digested. It is proposed to purchase a farm of 1000 acres in the Ozarks, and to stock it with goats procured from

Switzerland, France, and Germany. The plan includes the leasing of goats to families, that milk may be had fresh every morning, and furnishing the animals free of charge to the indigent.

---

ELISHA KENT KANE, physician, scientist, and explorer, says the *Philadelphia Medical Journal*, was honored on the occasion of the twenty-first anniversary of the founding of the school bearing his name, at Twenty-sixth and Jefferson streets, Philadelphia. Mrs. Thomas L. Kane, of Kane, Pa., presented to the school four water-color paintings by Mr. Kane, done while he was ice-bound on the expedition he led in relief of the Sir John Franklin party that had crossed the Arctic circle in search of the north pole. Amos Bonsal, the last survivor of the relief party, made an address.

---

THE population of the Island of Capri, says the *Medical Record*, is indignantly protesting against the plan of Professor von Behring to transform the villa which he bought from the family of the late Herr Krupp, the famous gunmaker of Essen, into a laboratory for the manufacture of antitoxin and a sanatorium for the treatment of people affected with tuberculosis. The people of Capri say that it would injure the reputation of the island, which depends for its revenues almost entirely on foreign visitors. They have interested several members of the Chamber of Deputies in their protest, and hope to be able to prevent the carrying out of Behring's project.

---

THE successful physician, says the *Northwestern Lancet*, is usually the man who attends strictly to his own business, and refrains from intruding into the affairs of others; who speaks kindly of his fellow practitioner, whether he deserves it or not; who is studious and observant, and profits by his own and the experience of others; who is full of good cheer, and is competent to give wholesome advice. If all physicians belonged to this school the medical profession would be a mighty power, capable of engineering mighty plans for the good of the people, united and respected, free from unjust criticism, and possessed of more than a "common mind."

---

A GERMAN physician, Schottelius, has been (says the *Journal of the American Medical Association*) carrying out some experiments on some chickens which are highly interesting, as tending to show that some bacteria are necessary to life. Five new-born chickens were given sterilized food, and though they ate much more than other chickens, none was able to live more than eleven to twenty-nine days. The digestion seemed to take place normally, but there was no increase in size. Schottelius made another experiment, which consisted in giving some of these chickens a culture of bacillus coli gallinarum. The chickens began to grow larger immediately, and no anomaly was noticed.

---

DUPLANT, of Lyons, a pupil of Professor Tripier, of Lyons, published in 1898 a work in which he declared that it was not proven that cancer ever succeeded ulcer of the stomach, and went so far as to say that such an event never took place. Dr. Andistere, according to a contemporary, has just published a thesis in which he proves that such a transformation may take place and is not rare. This is more frequently seen in ulcers situated in the prepyloric region, and begins by the borders of the ulcer. A diagnosis can be made out by the persistence of the morbid symptoms after prolonged treatment, especially the pain. The prognosis is much more grave than that of ordinary cancer, as complications of both diseases may supervene.

---

THE special committee appointed at Memphis to consider the question of meeting-place for 1904 has just reached a conclusion, and the thirtieth annual meeting of the Mississippi Valley Medical Association will be held at Cincinnati, O., October 11, 12, 13,

1904. Dr. B. Merrill Rickets has been elected chairman of the Committee of Arrangements. The following are the officers for the year: President, Edwin Walker, M.D., Evansville, Ind.; President-elect, Hugh T. Patrick, M.D., Chicago; First Vice-President, Bransford Lewis, M.D., St. Louis; Second Vice-President, Geo. W. Cale, Jr., M.D., Springfield, Mo.; Secretary, Henry Enos Tuley, M.D., Louisville, Ky.; Assistant Secretary, S. C. Stanton, M.D., Chicago; Treasurer, Thos. Hunt Stuckey, M.D., Louisville, Ky.

---

DR. OTTO SCHMIDT, of Cologne, according to the *Medical Record*, claims to have isolated the parasites of cancer, and says that all the alleged parasites of varied appearance described by Gaylord and others are only varieties of the same microorganism. He says he has obtained an effective antiserum, and has also been able to diagnose malignant diseases by means of a Widal reaction. The serum does not remove the growth, but takes from it its character of malignancy, and thus makes it possible for the surgeon to extirpate it without fear of recurrence. The discoveries of Schmidt were recently described by Dr. Jesse Johnson in a paper read before the Abernethian Society of London, but were received with considerable skepticism by the members of the society.

---

THE Army Medical School began its seventh annual session November 3 with twenty-seven student officers in attendance. The faculty consists of the following officers: Col. Chas. Heizmann, president of the faculty and professor of military medicine; Major William H. Arthur, professor of duties of medical officers in peace and war; Major William C. Borden, professor of military surgery; Major Walter D. McGraw, professor of military hygiene and lecturer on tropical diseases; Major James D. Glennan, ophthalmology and optometry; Captain F. P. Reynolds, instructor in hospital corps drill and medical department administration; Captain C. R. Darnall, secretary of faculty, instructor in sanitary chemistry and operative surgery; Lieutenant James Carroll, professor of bacteriology and clinical microscopy; and Captain H. G. Trout, instructor in equitation.

ACCORDING to the *British Medical Journal*, at Helsingfors, in Finland, medical practitioners, it is said, dictate their prescriptions to their patients through the telephone. The patient has to repeat the prescription after dictation, and each prescription is marked with a number corresponding to that under which it is entered in the practitioner's case-book. The system seems at first sight to be fraught with greater risks of evil consequences even than those which Mr. Idris has attributed to the dispensing of drugs by doctors. It is true the danger of fatal mistakes is minimized by a provision of the law which forbids the prescription of toxic substances by telephone. Nevertheless, there remain large possibilities of results to say the least unpleasant arising from this mode of prescribing.

---

THE plan of a summer camp for the insane, says the *New York Medical Journal*, has been successfully tried by Dr. Eugene H. Howard, superintendent of the Rochester Hospital, under direction of the State Commission in Lunacy. The camp was situated on the shore of Lake Ontario in the town of Webster, eight miles from Rochester. Only harmless and partly recovered patients were allowed to occupy the thirty tents erected on four acres of ground surrounding an old-fashioned cottage, with barns, and seventy-two men and one hundred and twenty-eight women were cared for with such excellent results that Dr. Howard has decided to make the innovation permanent. The demeanor of the patients, relieved from restraint, was carefully watched, and it was noticed that improvement was rapid.

---

LOUIS PASTEUR, the father of modern scientific medicine, has left as a heritage to humanity the great laboratories and hospitals in Paris compositely known as the Pasteur Institute. Here are made the various serums that are widely used in the Old World and that to a less extent are employed in America. The U. S. A. Government regulation of the manufacture and sale of all serums, both domestic and foreign, is praiseworthy paternalism, for it insures cleanliness, caution and care in every step of the process of making, handling and selling the various serum products. Each package of liquid serum bears a date-mark which indicates the extreme limit of its full therapeutic value. It is authoritatively stated that the serums in dry form that are made in the Pasteur laboratories keep indefinitely in any climate. If this be true these serums would be a valuable addition to our armamentarium.

---

WILLIAM M. WARREN, general manager for Parke, Davis & Co., died at his home in Detroit, Mich., November 11. Spinal meningitis was the immediate cause of death, but Mr. Warren had been in poor health for several weeks. He was just home from Hot Springs, Va. Wm. M. Warren was born at Smith Station, Ala., in 1864. He entered the New York office of Parke, Davis & Co. in 1880. His services were so satisfactory that in 1886 he was transferred to the Detroit office. His work in that place was in the laboratories. This was followed by experience on the road, and in 1892 he became assistant manager of the firm. In 1896, Geo. S. Davis, general manager, retired and Mr. Warren succeeded him at the head of the firm. The employees of the company have recently published a memorial leaflet which shows touching and affectionate regard for the high character of the man. He was publisher of the *Medical Age*, *Medicine* and *Therapeutic Gazette*.

---

AN increase in the death-rate of the army from 13.94 per 1,000 in 1901 to 15.49 per 1,000 in 1902 is shown in the annual report of Surgeon-General R. M. O'Reilly for the fiscal year ending June 30. This increase is, according to *American Medicine*, attributed to cholera, which caused 3.54 deaths per 1,000. A slight improvement appears in the hospital admission rate for disease and injury, which declined from 17.9159 per 1,000 in 1901 to 17.1651 per 1,000 in 1902. Discussing other features of the health of the army, Surgeon-General O'Reilly's report says: "The enrollment of about 5,000 native Filipino scouts having added a new racial element to the army, it becomes a matter of much interest to study the comparative effects of disease on them and on our white and colored troops. For the whole army at home and abroad during the year of 1902, the white troops showed an admission rate of

17.0033 per 1,000 and a death-rate of 14.40. The negro troops had 18.9774 admissions and 24.11 deaths per 1,000, and the Malay scouts 17.0721 admissions per 1,000 and 24.4 deaths. The white race, therefore, gave the lowest figure in sickness and much the lowest mortality. The black race led in both, although the Malay closely approached it in death-rate. The steadily increasing prevalence of venereal disease is the most discouraging feature in the sick report of the army."

---

THE report of the committee appointed by the Atlanta Society of Medicine to draft resolutions upon the death of Dr. James McFadden Gaston is as follows:

In the death of Dr. James McFadden Gaston, there has passed from our midst, one whose long and honorable services in our profession entitle him to especial remembrance. Full of days, and with the record of a well-spent life, he was the "Nestor" of the medical profession of the South, upon whose annals in the surgical world especially his eminent services in the course of original investigation and discovery have shed a particular lustre. He was indeed a most zealous and enthusiastic disciple of Hippocrates, illustrating the best traditions of the profession, from the time when, as a young surgeon and patriot, he carried its beneficent influence and healing calm on many bloody battle-fields of the Confederacy to later years, when undismayed by adversity and uncorrupted by want, he pursued the scientific investigations of that profession to which he had dedicated his whole soul and life. And although with him the "Almond-tree" did flourish, his active and fertile mind never grew old! Perhaps the most unusual and distinguished trait of his personality was that freshness and vigor of mind that seemed amidst the physical disabilities of age to rise superior to the material restrictions of the body. A most marked trait of his character was the kindly and respectful consideration and innate modesty of self-opinion with which he treated the younger brethren of the profession. Not to him did it occur to disparage merit on account of *youth*, and thus "damn with faint praise!" This, with his enthusiastic interest in the most advanced progress of his profession, is an impressive example to those who at half his span of years succumb to the mere material and selfish aspects of our noble profession. He may be truly said to have "died in harness," as until a few days of his end he was to be found in his office. A true scientist in his profession, a thorough gentleman in his life, he leaves behind him an impressive example for our emulation, and a memory that "smells sweet and blossoms from the dust."

Signed:

JOHN C. OLMSTED,
BERNARD WOLFF,
W. B. EMERY.

